# Prevalence of depressive symptoms among schoolchildren in Cyprus: a cross-sectional descriptive correlational study

**DOI:** 10.1186/s13034-017-0145-8

**Published:** 2017-02-02

**Authors:** Sokratous Sokratis, Ζilides Christos, Panagi Despo, Karanikola Maria

**Affiliations:** 1 Department of Nursing, Faculty of Health Sciences, Cyprus University of Technology, 15, Vragadinou Street, Limassol, Cyprus; 2 Department of Medicine and Epidemiology, Faculty of Health Sciences, Larissa University, Larissa, Greece; 3 Nursing Division, Community Mental Health Services, Limassol, Cyprus

**Keywords:** Children depression inventory (CDI), Depressive symptoms, Cyprus, Young, Self-esteem, Validity

## Abstract

**Background:**

Depressive symptoms in the young constitute a public health issue. The current study aims to estimate: (a) the frequency of depressive symptoms in a sample of final grade elementary-school children in Cyprus, (b) the association among frequency of depressive symptoms, gender and nationality and, (c) the metric properties of the Greek-Cypriot version of the children’s depression inventory (CDI).

**Methods:**

A descriptive cross-sectional study with internal comparison was performed. The occurrence of depressive symptoms was assessed with the CDI, which includes 5 subscales: depressive mood, interpersonal difficulties, ineffectiveness, anhedonia and negative self-esteem. Clinical depressive symptoms were reported as CDI score ≥19. CDI was anonymously and voluntarily completed by 439 schoolchildren [mean age 12.3 (±0.51) years old] from fifteen public elementary schools (217 boys and 222 girls), yielding a response rate of 58.2%. The metric properties of the CDI were assessed in terms of internal consistency reliability and construct validity via exploratory factor analysis (rotated and unrotated principal component analysis). Descriptive and inferential statistics were explored.

**Results:**

10.25% of Cypriot schoolchildren reported clinical depressive symptoms (CDI score ≥19). Statistically significant differences were reported between boys and girls in all five subscales of the CDI. Girls reported higher scores in “Depressive mood”, “Negative self-esteem” and “Anhedonia” subscales, while boys scored higher in “Interpersonal difficulties” and “Ineffectiveness” subscales. There were no statistically significant differences among ethnicity groups regarding the entire CDI or the subscales of it. Concerning the metric properties of the Greek-Cypriot version of the CDI, internal consistency reliability was adequate (Cronbach’s alpha = 0.84). Factor analysis with varimax rotation resulted in five factors explaining 42% of the variance.

**Conclusions:**

The Greek-Cypriot version of the CDI is a reliable tool for the assessment of the severity of depressive symptoms in schoolchildren. Institutional counseling services, as well as interventions aiming to empower the young need to address the different psychological needs of boys and girls. Longitudinal studies within this cultural context may be warranted, with special attention to other factors related to depressive symptoms and low self-esteem in schoolchildren, such as suicidality or bullying.

## Background

The occurrence of depressive symptoms in children and young people constitutes a serious public health issue [1], addressed by the WHO [2, 3]. It is recognized as a common, however unbearable, disturbance in these populations, affecting all areas of functioning, such as motivation, cognitive performance, emotions, mood, and perception of self-worth [4]. Moreover, depressive symptoms in schoolchildren may disrupt the course of life during a critical period for learning and social development [5]. Depressive symptoms may affect students of any age, gender, ethnicity and socio-economic status [6].

International population-based studies in children aged 7–13 years show that the occurrence of severe depressive symptoms ranges from 4% to as high as 26.1% [7–16]. Specifically, in an epidemiological research carried out in the USA, it was estimated that 20–46% of boys and 25–56% of girls in puberty, as well as 15–20% of boys in childhood would report depressive symptoms at some point in their life [17]. Moreover, wealth of published evidence shows that childhood and adolescence are periods of life associated with the onset of depressive episodes, and that there is a dose- response relationship. Other studies argue that the severity of the first episode of depressive symptoms, as well as the age of occurrence are both associated with the outcome of the episode [10–13, 15, 17, 18], making early screening of depressive symptoms in the young extremely important. However, there is scant data with regard to the prevalence of such symptomatology in elementary students [2], and, to the best of our knowledge, there is no such data in the Cypriot schoolchildren population, either. Additionally, depressive symptoms in the young have been shown to be associated with life-threatening behaviors, such as suicidal attempts or self-harming, with the female gender identified as a risk factor for the latter [19]. As a result, the WHO has declared the need for data on the prevalence of depressive symptoms in the young, particularly with regard to gender differences and cultural-related factors, so that the necessity for relevant intervention is illustrated [20, 21].

Nevertheless, there has been conflicting evidence regarding the association between gender and depressive symptoms among children and adolescents; some researchers report more than doubling of relevant symptomatology in boys as compared to girls [22–30], whereas others find girls to be more affected or to have equal rates with boys [10, 15, 31]. Additionally, the association between depressive symptoms and ethnicity in children is being debated. There has been literature suggesting a possible causal relationship between ethnicity and depressive symptoms in children [15, 31], while other researchers doubt that such an association exists [11, 32, 33].

### Aim

The current study aims to add evidence to existing literature by: (a) estimating the frequency of depressive symptoms in a sample of final grade elementary-school children in Cyprus, (b) exploring the association among frequency of depressive symptoms, gender and nationality, and (c) investigating the metric properties of the Greek-Cypriot version of the children depression inventory (CDI).

## Methods

### Study population and design

#### Design

A descriptive cross-sectional study with internal comparisons was performed in 2009 on a nationwide public-school-based sample of Greek-Cypriot children aged 11–13 in Limassol, Cyprus.

#### Sampling

**Fig. 1:**
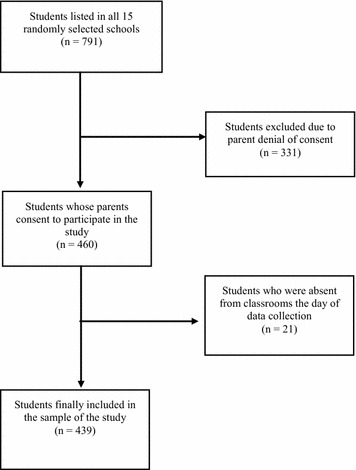
Respondents’ enrolment in the sample of the study

The total number of final-year attendees of public elementary schools in the metropolitan area of Limassol during the school year 2008/9 was 1536 [34]. Study sample estimation based on the tables of Cohen on detecting a moderate correlation effect, applying a statistical power of 80% and a level of statistical significance of 0.05, yielded a required sample of 308 schoolchildren. Initially, we decided to approach more than double the calculated sample-size, because we anticipated: firstly, parental refusal to offer their consent, and, secondly, the likelihood of schoolchildren being absent from class on the day of questionnaire filling. Fifteen schools were randomly selected from a list containing all 42 public elementary schools in the Limassol area. All 791 children cumulatively attending these schools were intended for study inclusion, irrespective of age, gender and ethnicity. As expected, a significant number of children were not included in the sample either because they were absent the day of recruitment (n = 21) or because parents declined to consent (n = 331). A final total of 439 children comprised the sample of the study (response rate 58.2%) (Fig. 1).

#### Data collection

Data collection was achieved through printed self-reported questionnaires. Each questionnaire comprised two parts. Part A included the demographic variables and Part B the CDI instrument for the assessment of depressive symptoms in schoolchildren. The questionnaires were distributed to children during class time (either in classrooms or labs). Prior to data collection, the primary investigator visited the selected schools in order to inform the principal and the teachers about the study. After this briefing, the primary investigator visited the classrooms explaining the aim of the study to the pupils in a comprehensive manner as well as distributing a sheet with the written explanation of it to them. The same sheet was given to parents by the primary investigator during pick-up time, when they had the opportunity to ask questions about the study. Then, parents were asked to return the form signed the day after, if they wished their kids to participate in the study. Reassurance was given that refusal to participate in the study would not have any consequences whatsoever for the schoolchild. Additionally, both parents had to sign the consent form. The day after, each teacher collected the signed forms, while a reminder and an extra week’s time was given for those who had not returned their forms. After a week, the primary investigator visited the school again and distributed the printed questionnaires to those students whose parents had eventually provided consent. The questionnaires were returned in a collection box in sealed envelopes to ensure anonymity. Data collection took place during a school period free of mid-term or final examinations or other potentially stressful, study-related activities.

#### Ethical approval

The study was approved by the National Bioethics Committee, as well as the Ethics Committee of the Ministry of Education of Cyprus.

### Instruments

#### Socio-demographic data questionnaire

Socio-demographic characteristics of the sample were assessed using a questionnaire specifically designed for the present study. This included individual characteristics (age, gender, and ethnicity).

#### The children’s depression inventory (CDI)

The children’s depression inventory (CDI) by Maria Kovacs [35, 36] is the most widely used and best studied instrument for the assessment of depressive symptoms in children. Previous research has confirmed the reliability and validity of the CDI in both clinical and non-clinical populations [35–37]. When administered to non-clinical population, the internal consistency reliability in terms of Cronbach’s alpha ranges from 0.76 to 0.88 [37], while its test-retest reliability has also been confirmed in previous studies [15, 35, 36]. With regard to the Greek version of the scale in schoolchildren, internal consistency reliability has been found 0.80 (Cronbach’s alpha), while split half reliability has been reported between 0.795 and 0.798 [15].

The scale comprises 27 items that quantify the severity of experienced depressive-related states, such as tearfulness, anhedonia, negative self-evaluation, suicidal thinking or hypochondriasis. For each item the respondent has three options with regard to the answers: 0: indicating absence of symptoms; 1: indicating mild symptoms; and 2: indicating severe symptoms. The total score ranges from 0 to 54. The 27 items are grouped into five subscales that correspond to the five major categories of depressive symptoms: (a) depressive mood, (b) interpersonal difficulties, (c) ineffectiveness, (d) anhedonia, and (e) negative self-esteem [35, 36].

Although this instrument has been validated in the Greek language in previous studies [15], it has not been used in the Cypriot population before, and particularly among children populations. As a result, the metric properties of the instrument had to be tested. The translation of the instrument into the Greek language followed relevant guidelines [37, 38]. The first step was a forward–backward–forward translation. The original English questionnaire was translated twice by two translators working independently. Then, all translated items were compared, in order to generate a single version for each item. The items were then translated back into English, so they could be compared with the items of the original English version. Thus, the final version of the Greek-language CDI questionnaire was produced.

### Data analysis

The Statistical Package for Social Sciences Software (SPSS-version 17) was used to analyze data. With regard to the metric properties of the CDI scale, internal consistency reliability was tested by Cronbach’s alpha coefficient and Guttman split-half alpha for the entire scale, while Cronbach’s alpha was also calculated for each of the five subscales. Additionally, item-to-scale and subscale-to-scale correlations by Pearson r coefficient were tested. The construct validity of the Greek-Cypriot version of the CDI scale was tested by exploratory factor analysis. Firstly, principal component analysis and unrotated factor solution were performed. The maximum-likelihood method was used for factor extraction [39]. Only factors that accounted for variances greater than 1 (eigenvalue >1) were included, and the number of factors was confirmed by examination of the scree plot. Further, the Varimax orthogonal rotation was used to minimize the number of variables that had high loadings on a factor, thus identifying meaningful factors. Following factor extraction, factor contents were tested by computation of internal consistency coefficients (alphas) [39].

Normality test and descriptive statistics of all variables were explored and mean values (M) and standard deviations (SD) were estimated. The severity of depressive symptoms, both overall (entire CDI) and components-related (CDI subscales) were calculated by summing the mean cumulative value of all entries in the rank-ordered questions. Floor and ceiling effects were calculated based on the percentages of scores at the extremes of the scaling range. Floor and ceiling effects were considered to be present when 15% of respondents had the minimum or maximum possible scores on a given dimension, respectively. Comparisons on categorical variables were carried out with the Chi square test, while the differences between the mean values of continuous data for different groups were investigated with the non-parametric Mann-Whitney U and Kruskal-Wallis tests in the case of variables not following the normal distribution. A cut-off point of 19 was introduced in order to identify children manifesting clinically relevant depressive symptoms, since, according to international literature, the score of 16–18 which had been used extensively as the cut-off point for the presence of clinically relevant depressive symptoms resulted in approximately 15–20% of false positive measurements, leading other researchers to suggest that a higher cut-off point may be used [35–37]. A significance level of 0.05 was applied in all comparisons.

## Results

### Demographic characteristics of the sample

The final sample consisted of 439 children, who successfully completed the data collection tool. Of these, 217 (49.4%) were boys and 222 girls (50.6%). Their mean age was 12.3 years (minimum value = 11, maximum value = 13; standard deviation = 0.51). All students (439) lived in metropolitan areas. The vast majority were of Cypriot origin (n = 390, 88.8%), while 49 students (n = 49, 11.2%) had different ethnicities.

### Scores in the CDI scale and subscales

The mean score [±Standard Deviation (SD)] in the entire CDI scale (overall mean score) for the study sample (n = 439) was 9.7 (±6.8) [scale range (SR): 0–54], which denotes non-clinically relevant depressive symptoms. The mean scores in each of the five subscales of the CDI scale are presented in Table 1. The highest mean score was reported in the Anhedonia subscale and the lowest in the interpersonal difficulties subscale.

**Table 1:** Mean scores in the CDI scale and subscales in the study sample (n = 439)

CDI scale & subscales	Minimum value	Maximum value	Mean value	Standard deviation	Standard error
CDI entire scale (27 items SR: 0–54)	0.00	34.00	9.7	6.8	0.1
Depressive mood subscale (6 items, SR: 0–12)	0.00	10.00	2.34	2.1	0.1
Inter-personal difficulties subscale (4 items, SR: 0–8)	0.00	6.00	0.95	1.0	0.1
Ineffectiveness subscale (4 items, SR: 0–8)	0.00	7.00	1.60	1.5	0.1
Anhedonia subscale (8 items, SR: 0–12)	0.00	13.00	3.01	2.5	0.1
Negative self esteem subscale (5 items, SR: 0–10)	0.00	8.00	1.84	1.7	0.1

*SR* scale range

With regard to the mean score of the entire CDI scale (overall mean score), there was no statistically significant difference across gender [CDI scores *boys* (n = 217): minimum value = 0, maximum value = 34; mean value = 9.23; standard deviation = 6.44] [CDI scores *girls* (n = 222): minimum value = 0, maximum = 32; mean value = 10.28; standard deviation = 7.13] Mann-Whitney U, p = 0.143). Similarly, no statistically significant differences were noted across ethnicity groups [CDI scores *Cypriots* (n = 390): minimum value = 0, maximum value = 34; mean value = 9.71; standard deviation = 6.78] [CDI scores *Others* (n = 222): minimum value = 0, maximum = 29; mean value = 10.18; standard deviation = 7.12] (Mann-Whitney U, p = 0.77).

### Frequency of clinically relevant depressive symptoms (CDI ≥ 19) and associations with gender and ethnicity

The frequency of clinically relevant depressive symptoms [CDI score ≥19] in the study sample (N = 439) was 10.25%, since 45 respondents (17 boys and 28 girls) scored 19 or above in the overall CDI. Although the frequency of clinically relevant depressive symptoms (CDI ≥ 19) was higher in girls and children of other ethnicities, both differences were not statistically significant [(x^2^ by gender, p = 0.099), (x^2^ by ethnicity, p = 0.137) (Table 2).

**Table 2:** Frequency of clinically relevant (CDI total score ≥19) and non-clinically relevant (CDI total score <19) depressive symptoms across gender and ethnicity groups in the study sample (n = 439)

	Total sample		Children reporting non-clinically relevant depressive symptoms		Children reporting clinically relevant depressive symptoms		X^2^	DF	p value
	N	%	N	%	Ν	%			
*Gender*							2.72	1	0.099
Male	217	49.4	200	92.2	17	7.8			
Female	222	50.6	194	87.4	28	12.6			
*Ethnicity*							2.21	1	0.13
Cypriot	390	88.8	353	90.5	37	9.5			
Other	49	11.2	41	83.7	8	16.3			

### Mean scores in the entire CDI scale and each CDI subscale in the group of respondents reporting clinically relevant depressive symptoms in the entire CDI scale (CDI ≥ 19) (n = 45)

The mean value in the entire CDI scale (overall mean score) in the group of schoolchildren who reported clinically relevant depressive symptoms (n = 45) (CDI ≥ 19) was 25.37 (minimum value = 19, maximum value = 34; standard deviation = 3.7). In this group of schoolchildren the most severe symptom in terms of intensity was anhedonia and the less severe regarded interpersonal difficulties (Table 3).

**Table 3:** Mean scores in the CDI scale and subscales in the group of participants with clinically relevant depressive symptoms (CDI ≥ 19) (n = 45)

CDI subscales	Minimum value	Maximum value	Mean value	Standard deviation	Standard error
Depressive mood subscale (6 items, SR: 0–12)	1.00	10.00	6.35	1.73	0.1
Inter-personal difficulties subscale (4 items, SR: 0–8)	0.00	6.00	1.95	1.38	0.1
Ineffectiveness subscale (4 items, SR: 0–8)	0.00	7.00	3.75	1.63	0.1
Anhedonia subscale (8 items, SR: 0–12)	3.00	13.00	7.40	2.55	0.1
Negative self-esteem subscale (5 items, SR: 0–10)	1.00	8.00	4.91	1.64	0.1

Although there was no statistically significant difference in the entire score of the CDI scale between boys and girls (Mann Witney-U, p = 0.395), as well as between Cypriot and non-Cypriot schoolchildren (Mann Witney-U, p = 0.341) in this group of respondents, however, statistically significant differences were noted between boys and girls in the mean scores in all five subscales of CDI scale (Table 4). The mean scores of clinically relevant symptoms in the “Depressive mood”, “Negative self-esteem” and “Anhedonia” subscales were statistically significantly higher in girls compared to boys (Mann Witney-U, p = 0.022–0.038). At the same time, boys exhibited statistically significantly higher mean score of clinically depressive symptomatology in the subscales “Interpersonal difficulties” and “Ineffectiveness” (Mann Witney-U, p = 0.034–0.042) (Table 4).

**Table 4:** Differences in the mean scores of each of the five CDI subscales in the group of schoolchildren with clinically relevant depressive symptoms across gender groups (CDI ≥ 19) (n = 45)

Subscales	Gender	N	Mean value	Standard deviation	p value
Depressive mood (6 items, SR: 0–12)	Boy	17	6.76	2.10	0.038
	*Girl*	28	8.1	1.44	
Inter-personal difficulties (4 items, SR: 0–8)	*Boy*	17	2.41	1.22	0.042
	Girl	28	1.67	1.41	
Ineffectiveness (4 items, SR: 0–8)	*Boy*	17	4.41	1.62	0.034
	Girl	28	3.35	1.54	
Anhedonia (8 items, SR: 0–16)	Boy	17	6.70	2.64	0.022
	*Girl*	28	7.82	2.45	
Negative self-esteem (5 items, SR: 0–10)	Boy	17	4.70	1.82	0.029
	*Girl*	28	6.03	1.55	

*N* number of respondents, *SR* scale range

### Metric properties of the child depression inventory (CDI)

With regard to the overall scoring on the entire CDI, it was found that Cypriot schoolchildren scored between 0 (floor = 3%) and 34 (3.5%), with no ceiling effect having occurred (total maximum score in the CDI scale was 54). As for the internal consistency reliability, the Cronbach’s alpha coefficient for the entire CDI scale was a = 0.845, and Guttman split-half alpha was 0.89; Cronbach’s alpha for the four out of 5 subscales of the CDI was 0.54 < a < 0.64 (Table 5). These values are in line with international literature, where a range of 0.71 to 0.89 for the entire CDI scale has been reported [36] while the subscale range has been reported as lower than 0.60 and higher than 0.57. Taking these into consideration, one could argue that the ‘Interpersonal difficulties’ subscale exhibited a relatively low internal consistency reliability in terms of Cronbach’s alpha coefficient (a = 0.32) compared to existing literature [36]. Indeed, in this subscale the item 12 exhibited low loading with the rest of the items in factor analysis, while at the same time it exhibited stronger loading with the anhedonia subscale, as well as with the depressive mood subscale (Table 6). C5 Furthermore, no difference was noted in the loading of item 12 with regard to gender groups.

**Table 5:** Internal consistency reliability of the five CDI subscales in terms of item-to-scale correlations (Pearson’s r) and Cronbach’s alpha coefficient

CDI subscales	Cronbach’s alpha	Item-to-scale correlation within each subscale (Pearson’s r)	Subscale-to-scale correlation (Pearson’s r)
Depressive mood	0.64	0.58–0.66* (0.52–0.61)*	0.84* (0.68*)
Inter-personal difficulties	0.32	0.17–0.33* (0.13–0.29)*	0.50* (0.36*)
Ineffectiveness	0.54	0.43–0.50* (0.44–0.46)*	0.64* (0.47*)
Anhedonia	0.64	0.59–0.64* (0.53–0.60)*	0.84* (0.64*)
Negative self-esteem	0.62	0.48–0.62* (0.45–0.5)*	0.78* (0.63*)

The values in the parenthesis regard uncorrected values (items excluded from subscales/subscales excluded from the CDI scale)

* p < 0.0001

**Table 6:** Component matrix of principal component analysis with the varimax rotation method (Kaiser normalization applied)

Items of the CDI scale	Component				
	1	2	3	4	5
Item 1 (sadness)	*0.641*	0.190		0.195	
Item 2 (pessimism)	0.256	*0.526*			0.190
Item 3 (self-inefficacy)		0.284		*0.482*	0.171
Item 4 (anhedonia)	0.175	0.245	*0.391*		
Item 5 (negative self-concept)		0.174		0.279	*0.531*
Item 6 (worryingness)	*0.625*	0.193			
Item 7 (self-acceptance	0.378	*0.591*			
Item 8 (self-blaming)	*0.123*	0.467		0.270	0.339
Item 9 (suicidal thinking)	0.263	*0.255*	0.177	0.128	0.229
Item 10 (tearfulness)	*0.577*	0.293			
Item 11 (irritability)	*0.506*	0.396			
Item 12 (socializing)	0.229		0.210		*0.002*
Item 13 (indecisiveness)	*0.125*	0.189			
Item 14 (self-image)	0.216	*0.670*	0.185		−0.139
Item 15 motivation)	0.173			*0.663*	
Item 16 (sleep)	0.488		*0.187*	0.330	
Item 17 (tiredness)	0.493	0.208	*0.193*	−0.136	0.266
Item 18 (appetite)	0.515	−0.137	*0.220*	0.163	
Item 19 (hypochondriasis)	0.451		*0.165*		
Item 20 (loneliness)	0.325	0.331	*0.547*		
Item 21 (school enjoyment)	0.125		*0.543*	0.235	
Item 22 (friendship)		0.254	*0.697*		
Item 23 (school performance)			0.244	*0.677*	
Item 24 (self-esteem)		0.531		*0.416*	
Item 25 (feeling loved)		*0.569*	0.380		
Item 26 (submissiveness)					*0.825*
Item 27 (inter-personal difficulties)			0.607	0.186	*0.277*

With italics are marked the items grouped in each factor according to the constructors of the scale [35, 36]. Factor loadings lower than 0.10 have not been reported herein

Furthermore, the item-to-scale correlations regarding the entire CDI (27 items) are presented in Table 7. The Pearson’s r coefficient ranged between 0.10 and 0.52. The item-to-scale correlations, as well as the subscale-to-scale correlations in each subscale are presented in Table 5,. With regard to subscale-to-scale correlation, statistically significant positive moderate to strong correlations were observed (0.36 < r < 0.68, p < 0.01). In Tables 5 and 7 we present both corrected (item excluded from the scale), and uncorrected (item included in the scale) correlational values.

**Table 7:** Item-to-scale correlations with Pearson’s r values in the CDI instrument

CDI items	Depressive-related state of each item	Item-to- scale correlation (Pearson’s r)
Item 1	Sadness	0.56* (0.51*)
Item 2	Pessimism	0.51* (0.45*)
Item 3	Self-inefficacy	0.42* (0.37*)
Item 4	Anhedonia	0.43* (0.37*)
Item 5	Negative self-concept	0.32* (0.27*)
Item 6	Worryingness	0.53* (0.46*)
Item 7	Self-acceptance	0.57* (0.52*)
Item 8	Self-blaming	0.46* (0.39*)
Item 9	Suicidal thinking	0.40* (0.35*)
Item 10	Tearfulness	0.52* (0.47*)
Item 11	Irritability	0.60* (0.53*)
Item 12	Socializing	0.29* (0.23*)
Item 13	Indecisiveness	0.34* (0.26*)
Item 14	Self-image	0.53* (0.46*)
Item 15	Motivation	0.38* (0.27*)
Item 16	Sleep	0.50* (0.43*)
Item 17	Tiredness	0.48* (0.40*)
Item 18	Appetite	0.45* (0.36*)
Item 19	Hypochondriasis	0.35* (0.27*)
Item 20	Loneliness	0.55* (0.50*)
Item 21	School enjoyment	0.39* (0.33*)
Item 22	Friendship	0.44* (0.39*)
Item 23	School performance	0.43* (0.37*)
Item 24	Self-esteem	0.48* 0.41*)
Item 25	Feeling loved	0.53* (0.47*)
Item 26	Submissiveness	0.18* (0.10*)
Item 27	Inter-personal difficulties	0.40* (0.35*)

The values in the parenthesis regard corrected values (items excluded from the CDI scale in the correlation between item and CDI total scale)

* p < 0.0001

With regard to the construct validity of the scale, exploratory factor analysis of the principal components, unrotated, produced 7 factors that explained 50% of the observed variance. Therefore, it was deemed appropriate to proceed with the rotated solution. The aim was to confirm the clustering of items into factors which should represent the constructs represented in the subscales [39]. The findings confirmed the construct validity of the 27-item CDI scale. The maximum likelihood factor analysis with Varimax rotation resulted in 5 factors that accounted for the 41.05% of the variance (Factor 1: eigenvalue 2.97, 11.0% of variance; Factor 2: eigenvalue 2.74, 10.1% of variance; Factor 3: eigenvalue 2.21, 7.80% of variance; Factor 4: eigenvalue 1.81, 6.73% of variance; Factor 5: eigenvalue 1.44, 5.33% of variance). The extracted factors fell into five groups which reflected the five dimensions of the CDI in line with the reports by the constructors [35, 36]. Minimal cross-loading of variables occurred, and where this was the case it reflected closely associated concepts. Groupings of factors were further refined through reliability analysis. The results supported the original categorization into subscales as specified by Kovacs [35, 36], except for the Inter-personal difficulties subscale in which the item 12 was poorly grouped with the rest of subscale items during factor analysis.

## Discussion

### The frequency of clinically relevant depressive symptoms in the sample

Since the primary scope of the present study was to estimate the prevalence of clinically relevant depressive symptoms in schoolchildren in Cyprus and possible associations with gender and ethnicity, a main finding herein was that approximately 10%, of the respondents reported such symptoms, suggesting that one out of ten participants in our sample might need formal mental health assessment. This frequency is in line with the majority of studies conducted in European countries and the USA on the subject [8, 10, 14, 36, 40]. The consistency of related research findings supports the notion of universality of the phenomenon under study and may, to one extent, be attributed to the fact that the same diagnostic tool, the CDI, was used in most studies. With regard to European countries, in a study conducted in Italy, the rate of clinically relevant depressive symptoms amongst children was 10.6% [8], whilst researchers in Spain similarly reported a rate of 11% [10]. In a Swedish study the rate was, again, 10% [40], as it was in a study from Estonia (9.96%) [13]. Researchers in England described a comparable range of 8–10% [16], too. A study in Greece, however, reported a rather wide (and somewhat lower) range of frequencies (4.4–14.96%), probably because a cut-off point in the CDI scale higher than 19 was employed to mark clinically relevant depressive symptoms [15]. Another Greek study found a higher range of rates (8.6–21.9%), again due to different CDI cut-off point (≤10 to 18) employed [41]. In the USA the rate of depressive symptoms in schoolchildren was found between 10 and 12% [14, 35]. At the same time, the frequency of clinically relevant depressive symptoms, using the CDI tool, was found higher in the Japanese [42], Arab [43], Thai [44], Korean [14] and Russian children populations, ranging from 14 to 26% [16]. This controversy, to some extent, may be the result of diverse: (a) methodological approaches, (e.g. population-based or clinical sample, the time the measurement took place, the different cut-off point in the tool used), and (b) socio-cultural characteristics and religious beliefs of the different target populations.

Despite the fact that our findings regarding the rate of clinically relevant depressive symptoms were in line with those in the international literature, one has to underline their importance, since this is the first study, to our knowledge, that tackled this issue in Cyprus. Generally, there is limited data regarding the rate of depressive symptoms in the general population in Cyprus [45], as well as in adolescents; there is only one study conducted in young adults, in particular university students [46, 47]. According to those research findings, the prevalence of clinically relevant depressive symptoms was 27.9%. Moreover, it was demonstrated a strong positive association between depressive symptoms and individual, parental, academic and health-related behavior characteristics, as well as a positive association between depressive symptomatology and the number and severity of stressful life events [46, 47].

It seems that the rate of clinically relevant depressive symptoms in schoolchildren is lower than the observed rates in university students in Cyprus [46, 47]. This suggests, on the one hand an increase in the frequency of depressive symptoms in early adulthood, and on the other the necessity for interventions targeted on the early diagnosis and management of such symptoms in childhood and adolescence. Nevertheless, the presence of depressive symptoms in schoolchildren at an early stage of their life may have a negative impact on their physical and mental well-being [48, 49]. Evidence shows that the presence of depressive symptoms in pre-adolescents is associated with self-harm, suicidal thinking and suicide attempts [50], as well as with higher risk for development of mood disorder in later adulthood [10, 13, 15, 48, 51]. Overall, preschool children, up to 5 years old, exhibit lower incidence of depressive symptoms compared to school-age children (6–12 years old), whereas adolescents display the highest incidence [13, 29, 30]. Therefore, early detection and effective treatment of depressive symptoms in schoolchildren may reduce the mental health burden on this population, as well as the future risk for mood disorder or depression. Moreover, early screening of depressive symptoms may add to the improvement of learning ability, productivity, interpersonal relationships, academic performance and quality of life in children [1–5].

In line with the above, future longitudinal studies are proposed, aiming to explore particular life-related factors in childhood that may lead to depressive symptoms. And, since comparisons among cross-sectional surveys conducted in different cultural settings are difficult, the need is for collaborative international studies to investigate the frequency of depressive symptoms among children populations across different settings and cultures by employing standard methodology, as well. In addition, future studies in Cyprus need to address the prevalence of clinically relevant depressive symptoms in other age subgroups among the young, as well as possible associations of it not only with individual, parental, academic and health-related behavior characteristics, but also with life-threatening behaviors, such as self-harming, substance misuse or risky driving [52].

### Differences in the frequency of clinically relevant depressive symptoms in relation to students’ gender and ethnicity

In the present study, no statistically significant differences were noted in the overall rate of depressive symptoms regarding gender. In contrast, there were cross-gender differences in the type of reported depressive symptoms, since girls reported higher rates in the “Depressive mood”, “Negative self-esteem” and “Anhedonia” symptoms, while boys exhibited a higher frequency in “Inter-personal difficulties” and “Ineffectiveness” related symptoms. This finding illustrates the diversity of manifestation of depressive symptoms between males and females, thus the necessity for gender sensitive interventions [53]. Research findings in relation to the above issue are contradictory, since some reveal a difference in the prevalence of depressive symptoms between males and females, while others report no gender discrepancies [13, 15, 35, 36, 54–62]. Variations in these findings may be attributed not only to differences related to the way these symptoms are experienced, but also in the way they are reported, with females being more likely to describe mood-related issues, such as sadness or negative self-image, while boys are more likely to describe behavior- related symptoms, such as fights or difficulties in school performance [6, 61].

Nevertheless, the reports by WHO do demonstrate higher frequency of psychopathology in females [62]. Generally, a higher rate of depression amongst females has been associated with socio-cultural characteristics, including biological and psychological factors [49, 63]. Thus, future longitudinal studies, national or international, aimed at larger pre-adolescent populations are recommended as essential to determine whether or not gender is an important factor affecting the manifestation of psychiatric morbidity.

With regard to ethnicity, the present study did not find significantly different rates of depressive symptoms, which is in line with existing literature [16, 31, 51]. Thus, further studies aiming to determine whether ethnicity is indeed an important factor are proposed [64].

### Validation of the child depression inventory (CDI)

Since the exploration of the metric properties of the CDI scale was included in the objectives of the present study, useful data allowing comparisons between different cultural contexts in terms of reliability and validity of the instrument have been produced. In particular, the present findings confirmed the internal consistency reliability of the Greek-Cypriot version of the CDI for elementary-school Cypriot children population, since Cronbach’s alpha coefficient for the entire CDI was comparable to the one reported previously in Greek schoolchildren [15], as well as international literature [37]. Additionally, in Cronbach’s alpha measures, we have to underline the relatively low internal consistency reliability of the “Inter-personal difficulties” subscale, a finding which was also reported in the factor analysis. Similar findings about this subscale have been previously reported in the literature [35, 36]. Accordingly, we suggest further exploration of the items included in this subscale through qualitative studies [35, 36, 65]. Overall, factor analysis confirmed the five dimensions of the CDI reported previously [35, 36].

Finally, one might advocate for a comparison of the CDI scale with other tools designed to assess depressive symptoms or with clinical psychiatric diagnostic interviews, so that additional metric properties such as discriminant validity, are tested [37].

## Limitations

The above findings need to be viewed in the context of certain methodological limitations. Although we investigated clinically relevant depressive symptoms in association with gender and ethnicity, the cross-sectional design of the present study does not allow for assumptions to be made on causality. What our findings do suggest is that further longitudinal studies should aim to explore gender influence along with other life stressors with regard to the manifestation of mild psychiatric symptoms. Furthermore, although the results of this study are based on a large and random sample of schoolchildren drawn from the general population, several factors limit the generalization of findings. Firstly, only urban schools were included in the study. The degree to which findings would differ if schools from rural areas of Cyprus had also been included is unknown. Secondly, the study was based on a sample of children who attended school, therefore it did not include children who had left or never attended school, including children of other ethnicity or of minorities (e.g. roma). Moreover, the absence of statistical difference in depressive symptoms between Cypriot children and those of other ethnicities could be attributed to the relatively small proportion of children coming from ethnic minorities.

Finally, both, the fact that a number of parents refused to consent for their children to participate in the research as well as the element that data collection took place during specific schooldays when a number of chosen students happened to be absent, may be considered as limitations.

## Conclusions

Elementary schoolchildren in Cyprus exhibited prevalence of clinically relevant depressive symptoms in concord with existing literature and the Greek-Cypriot version of the CDI proved to be a reliable tool for that assessment. Institutional counseling services, including strategies for screening and effective stress-management, together with interventions aiming to empower schoolchildren, have to take into consideration the different psychological needs of boys and girls. Adaptive coping strategies (i.e. cognitive flexibility, strategy-situation fit, and goal attainment) have been found to be associated with higher levels of positive adjustment [66] and lower levels of depressive symptoms; still, they need to be implemented according to the specific needs of the target populations. Further investigation with longitudinal studies within this particular cultural context may be warranted, addressing additional variables related to depressive symptoms, such as self-harming, suicidality or different types of bullying [50].
